# Obesity-Related Epigenetic Changes After Bariatric Surgery

**DOI:** 10.3389/fendo.2019.00232

**Published:** 2019-04-16

**Authors:** Andrea G. Izquierdo, Ana B. Crujeiras

**Affiliations:** ^1^Epigenomics in Endocrinology and Nutrition Group, Instituto de Investigacion Sanitaria (IDIS), Complejo Hospitalario Universitario de Santiago (CHUS/SERGAS), Santiago de Compostela, Spain; ^2^CIBER Fisiopatologia de la Obesidad y Nutricion (CIBERobn), Madrid, Spain

**Keywords:** DNA methylation, epigenetic reprograming, metabolic balance, non-coding RNA (ncRNA), weight loss

## Abstract

**Objective:** In recent years, an increasing number of studies have begun focusing on epigenetics as a link between environmental factors and a greater predisposition to the development of obesity and its comorbidities. An important challenge in this field is the evaluation of the possibility of the reversal of obesity-related epigenetic marks by means of therapy to induce weight loss and if the beneficial effects of therapy in reducing obesity are mediated by epigenetic mechanisms. We aimed to offer an outline of the current results regarding to the impact of bariatric surgery on epigenetic regulation, as well as to show if the beneficial effect of this intervention could be mediated by epigenetic mechanisms.

**Methods:** A review of the scientific publications in PubMed was performed by using key words related to obesity, epigenetics and bariatric surgery to provide an update of recent findings in this area of research. The most relevant and recently published articles and abstracts were selected to frame this review.

**Results:** Previous studies have demonstrated the presence of differential DNA methylation after bariatric surgery and the differential expression of non-coding RNAs. Therefore, epigenetic regulation could mediate the benefit of bariatric surgery on body weight and the metabolic disturbances associated with excess body weight, such as insulin resistance, hypertension, and cardiovascular disease. This evidence is relatively new as epigenetic regulation was first evaluated in the obesity field only a few years ago. However, there is an urgent need to perform longitudinal studies to evaluate the capacity of epigenetic marks in the prediction of bariatric surgery response.

**Conclusions:** Bariatric surgery appears to be capable of partially reversing the obesity-related epigenome. The identification of potential epigenetic biomarkers predictive for the success of bariatric surgery may open new doors to personalized therapy for severe obesity.

## Introduction

Obesity is currently a huge healthcare problem, worldwide, and is a risk factor for several diseases such as type 2 diabetes (T2D), cardiovascular disease and cancer (1). As the prevalence of obesity reaches pandemic proportions, this metabolic disease is estimated to become the biggest cause of mortality in the near future (2). In fact, it is extremely alarming, given that 2.3% of men and 5.0% of women have a body mass index (BMI) ≥35 kg/m^2^. The worldwide prevalence of morbid obesity was reported to be 0.64% in men and 1.6% in women (3).

Obesity is a multifaced chronic disease, the cause of which is a disbalance between energy consumed and the energy burned off. This disbalance is promoted by several factors such as unhealthy diet, sedentarism, and genotype (4). As consequence, surplus energy is stored in the adipocytes, and leading to an adipose tissue dysfunction characteristic of obesity, as well as other metabolic disorders, such as alterations in insulin sensitivity, blood pressure and the plasma lipid profile, which are risk factors that together define metabolic syndrome (5). Abdominal obesity is a powerfully risk factor of cardiovascular disease and T2D, independently of BMI (6).

Many of the aforementioned metabolic alterations occur as a result of an interplay between environmental, lifestyle and genetic factors (7). Metabolic diseases are strongly affected by physical inactivity and unhealthy dietary habits (8). Disappointingly, regardless of exhaustive genetic research on these disorders, the molecular mechanisms are scarcely elucidated. However, recently, it has been proposed that epigenetic mechanisms may be one factor underlying the development of the metabolic syndrome. Especially, there has been an growing interest in the potential role of epigenetics in the development of metabolic diseases, and in how lifestyle habits are associated with these changes (8).

In terms of weight-loss therapy, lifestyle changes such as those focusing on diet and exercise have poor long-term adherence, while pharmacological interventions are scarce.

Bariatric surgery is indicated when a patient shows a BMI higher than 40 kg/m^2^ or higher than 30 kg/m^2^ if obesity I coexisting with other comorbidities such as diabetes mellitus. The techniques of bariatric surgery are divided into restrictive and malabsortive or a combination of both. Among the most widely used techniques are the sleeve gastrectomy and the adjustable gastric band which are restrictive and the Roux-en Y gastric bypass (RYGB) which is a combined malabsorptive and restrictive procedure and it is the most used and most studied procedure. Other procedures such as the biliopancreeatic diversion are used with less frequency (9). Bariatric surgery is currently the most effective intervention (10), however, a significant number of patients are predisposed to recover the weight lost after surgery.

To underlying the mechanisms involved in this unsuccessful outcome and to find predictive biomarkers is a challenge for the obesity physicians (11). Additionally, the molecular mechanisms associated with the beneficial effect of bariatric surgery are still unclear. An important challenge in this field is to evaluate if it is possible to reverse the obesity-related epigenetic marks by means of therapy to induce weight loss and if the beneficial effects of therapy to reduce obesity are mediated by epigenetic mechanisms. Understanding the association between epigenetic mechanisms in obesity management could provide targets for personalized therapy.

In this review, we offer a snapshot of the current results in the epigenetics research field and bariatric surgery, as well as the impact of this intervention on epigenetic profiles in the counteraction of obesity and its comorbidities, and to predict the individual responses to this therapy.

## Mechanisms Involved in Epigenetic Regulation

The genetic information is the same in all the types of cells present in an organism, although their functions and characteristics are different. The mechanisms responsible for this cellular differentiation that originate the different phenotypes are the epigenetic marks of the genomes. The concept of epigenetics was first defined by Conrad Waddington in the early 1940s. He defined epigenetics as “the branch of biology that studies the causal interactions between genes and their products that give rise to the phenotype” (12). In later years, this definition was elaborated upon and is nowadays commonly accepted as “the study of changes in genetic function that are mitotically and/or meiotically inheritable and that do not imply a change in the DNA sequence” (13). The genome of an organism is generally unchanging. In contrast, the epigenome can be altered by the effect of external environmental factors (including lifestyle, nutrient intake, stress, exposure to toxins, physical activity, medical history, etc.), allowing in a short-time to provide a dynamic response by the organism (14). Therefore, epigenetics may explain why many different cell types are generated during an organism development, in spite of containing the same genetic information (15). Hence, epigenetic investigations aim to understand how external factors regulate gene expression, and even phenotypic traits (16).

**Figure 1:**
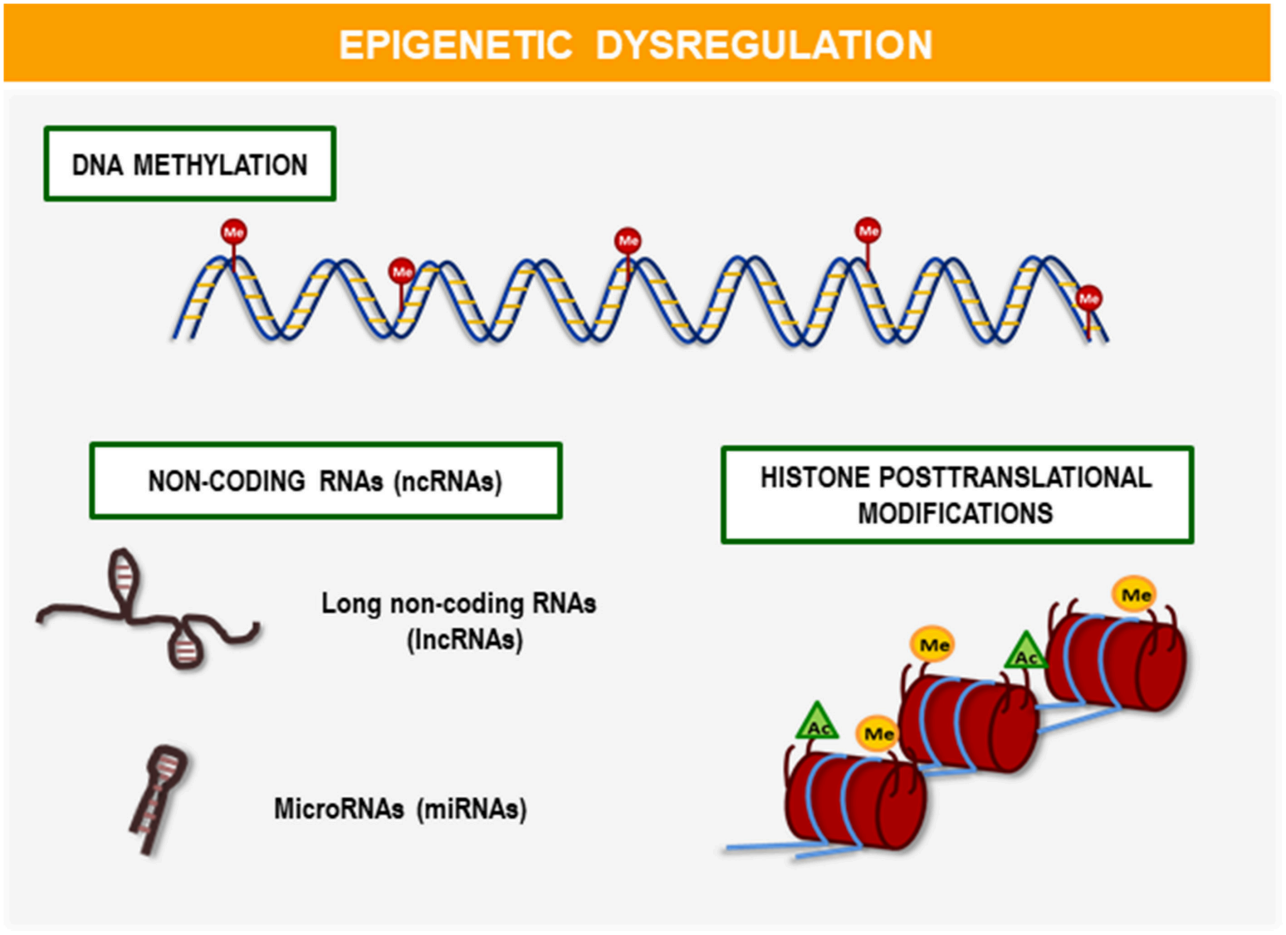
Mechanisms of epigenetic regulation. The main mechanisms involved in epigenetic regulation are: DNA methylation, histone post-translational modifications (PTMs), and non-coding RNAs (ncRNAs) including microRNAs (miRNAs), and long non-coding RNAs (lncRNAs). These epigenetic modifications play a fundamental role in the development and regulation of gene expression throughout life. Recent studies demonstrate its relevance in clinical application as biomarkers or potential therapeutic targets for the early diagnosis and personalized treatment of diseases such as obesity and its comorbidities.

Epigenetic markers are chemical modifications mediated by DNA enzymes and their chromatin proteins that play a key role in the regulation of genomic functions. The main mechanisms that lead this epigenetic regulation are (Figure 1): DNA methylation, histone post-translational modification (PTM), and non-coding RNAs (ncRNAs) including microRNAs (miRNAs) and long non-coding RNAs (lncRNAs) (15). Among these mechanisms, DNA methylation is the most abundant in the organism and the most known.

### DNA Methylation

Methylation corresponds to the biochemical addition of a methyl group to a molecule. In DNA from mammals, DNA methyltransferase enzymes (DNMTs) catalyzed this biochemical process and predominantly occurs in CG dinucleotides. The regions with a high density of CG dinucleotides, often found in genes in the promoter region, are referred to as CpG islands (17, 18).

DNA methylation can be evaluated in different genomic contexts: transcriptional start sites with or without CpG islands, in gene bodies, at regulatory elements and at repeat sequences (19). In general, CpG island methylation status has been correlated with transcription regulation, wherein methylated CpG islands in the promoter region are located upstream of switch off genes and unmethylated CpG islands are located upstream of switch on genes (20). While promoter methylation is associated with a decrease expression, it is known that intragenic methylation (gene body, 5′UTR and 3′UTR) is related to increased gene expression (19).

As DNA methylation is modulated by environmental and lifestyle factors, this is an important mechanism underlying metabolic alterations (21). Different processes can be involved in the influence of lifestyle factors on the regulation of the epigenetic machinery (22). There is increasing evidence stating that lifestyle changes, including weight loss, can have an impact on DNA methylation and consequently in gene expression (23). Therefore, an important function of DNA methylation is to drive the gene expression (24). There are multiple types of epigenetic modification, and each type has a fundamental role in the development and regulation of gene expression throughout one's life course (18). In general, epigenetic marks are stable, although all levels of epigenetic modifications can be reverse. Specifically, DNA methylation can change from high levels to low levels of methylation and vice versa. This process can be promoted by different players, such as epigenetic drug intake [for example, the hypomethylating agent−5-aza-2′-deoxycytidine (5-aza-dC)] and healthy lifestyle habits (25). A dietary treatment with functional foods and exercise can modulate DNA methylation and as consequence, the gene expression leading to an improvement of a specific diseases phenotype (17, 26).

The assessment of DNA methylation has strong applicability to disease management (27). It is possible to identify biomarkers for obesity and other related diseases, which serve as predictors of an specific biological status for personalized medicine (28). Traditionally, DNA methylation studies were aimed on quantifying the methylation level of the target genes and determining the total level of 5-methyl-cytosine (5mC) (29). With the use of microarray hybridization technology, it is possible to measure the genome-wide DNA methylation. With the next-generation sequencing platforms genomic maps of DNA methylation can be built at a single-base resolution (30).

Greatest resolution and precision is obtained when DNA methylation is evaluated by bisulfite conversion and then sequenced (31). By means of the bisulfite treatment, DNA cytosines pass to uracil, but only when the cytosine is unmethylated. Thus, the sequencing assessment is able to distinguish methylated and unmethylated cytosines can be distinguished by sequencing (32).

### Histone Post-translational Modification (PTM)

The post-translational modifications of histones play an important role in the control of gene expression, demonstrating their clinical relevance. Therefore, the number of investigations in this field has increased in recent years.

Histones are the main components of chromatin, a protein that together with DNA integrates chromosomes. Histones can be post-translationally altered by methylation, phosphorylation, acetylation or ubiquitination. These chemical processes are associated with the restructuring of chromatin, producing a more or less compacted state that activates (euchromatin) or inactivates (heterochromatin) the transcription of DNA (33). This modifications can occur at different sites simultaneously and the regulation between histone modifications can ensue within the same site, among different histone tails of in the same histone tail (15). Current studies have demonstrated the effect of histone modifications in adipogenesis (34, 35) and obesity (36, 37).

### Non-coding RNAs (ncRNAs)

ncRNAs have arisen as relevant transcriptional factors in physiological and pathophysiological circumstances; therefore, gene silencing with their use can be employed as a therapeutic strategy. There are different types of ncRNAs in human genome as it was shown by whole-genome studies (38).

First, microRNAs (miRNA) are small, evolving, single-stranded and non-coding RNA molecules that represent 1–5% of the human genome and regulate at least 30% of the genes encoding proteins. Although little is currently known about biological functions, it is apparent that they are decisive players in the gene expression regulation that controls various cellular and metabolic pathways (39). MiRNAs are being increasingly described as having the capacity to impact alterations in the metabolism. The potential of miRNA as therapeutic targets, as well as disease markers to fight obesity has been highlighted (40, 41).

Additionally, long non-coding RNAs (lncRNAs) also represent a new emerging class of ncRNAs (17). For a long time, they were considered as the transcriptional “noise” of the genome; however, they have received considerable attention in recent years. LncRNAs are defined as RNA molecules with more than 200 nucleotides that present in large numbers in genome and they are involved in chromatin remodeling, as well as transcriptional and post-transcriptional regulation (42). Several lncRNAs have been identified for their role in the regulation of adipogenesis (white adipose tissue and brown adipose tissue) and energy metabolism (43). Therefore, their use as potential therapeutic targets to combat obesity is currently being considered.

## Epigenetics IN Obesity and Comorbidities

There is growing interest on epigenetics in the scientific community and its role in the development of chronic diseases such as obesity. The reason for this recent interest lies in the current knowledge that metabolic diseases are highly associated with epigenetic alterations. In fact, in the last two decades, scientists have generated a variety of valuable data and knowledge based on epigenetic and cellular metabolism studies (16). Recently, it has been claimed that epigenomes may represent the molecular bridge connecting gene regulation and environmental factors in defining the risk of obesity. Furthermore, it was hypothesized that epigenetic dysregulation could contribute to a rapid increase in the prevalence of obesity and its complications due to the heritability of acquired epigenetic marks (44).

Mostly of recent findings in the area of epigenetics and obesity research have been provided by human studies. These studies include those that investigate the relationship between total methylation, site-specific methylation or whole-genome methylation and obesity, as well as studies examining the impact of interventions on DNA methylation profiles and obesity (45).

### DNA Methylation in Obesity

Differences in DNA methylation related to obesity or dietary interventions and weight loss use to be lower than 20%, even though this depend on the condition and tissue to be studied. A major limitation in many human studies is that epigenetic marks used to be measured in blood cells (as it is readily accessible for DNA analyses), instead of target tissues of metabolic disorders. Regardless of this, new studies have demonstrated the association between specific methylation sites in both blood and adipose tissue. According to this, Dick et al., demonstrated the association between DNA methylation levels and body mass index, the results in adipose tissue were reflected in whole blood (46). This warrants the use of DNA methylation profiling of blood cells for the detection of relevant epigenetic changes and offers a motivation for other appropriate studies (46, 47).

**Figure 2:**
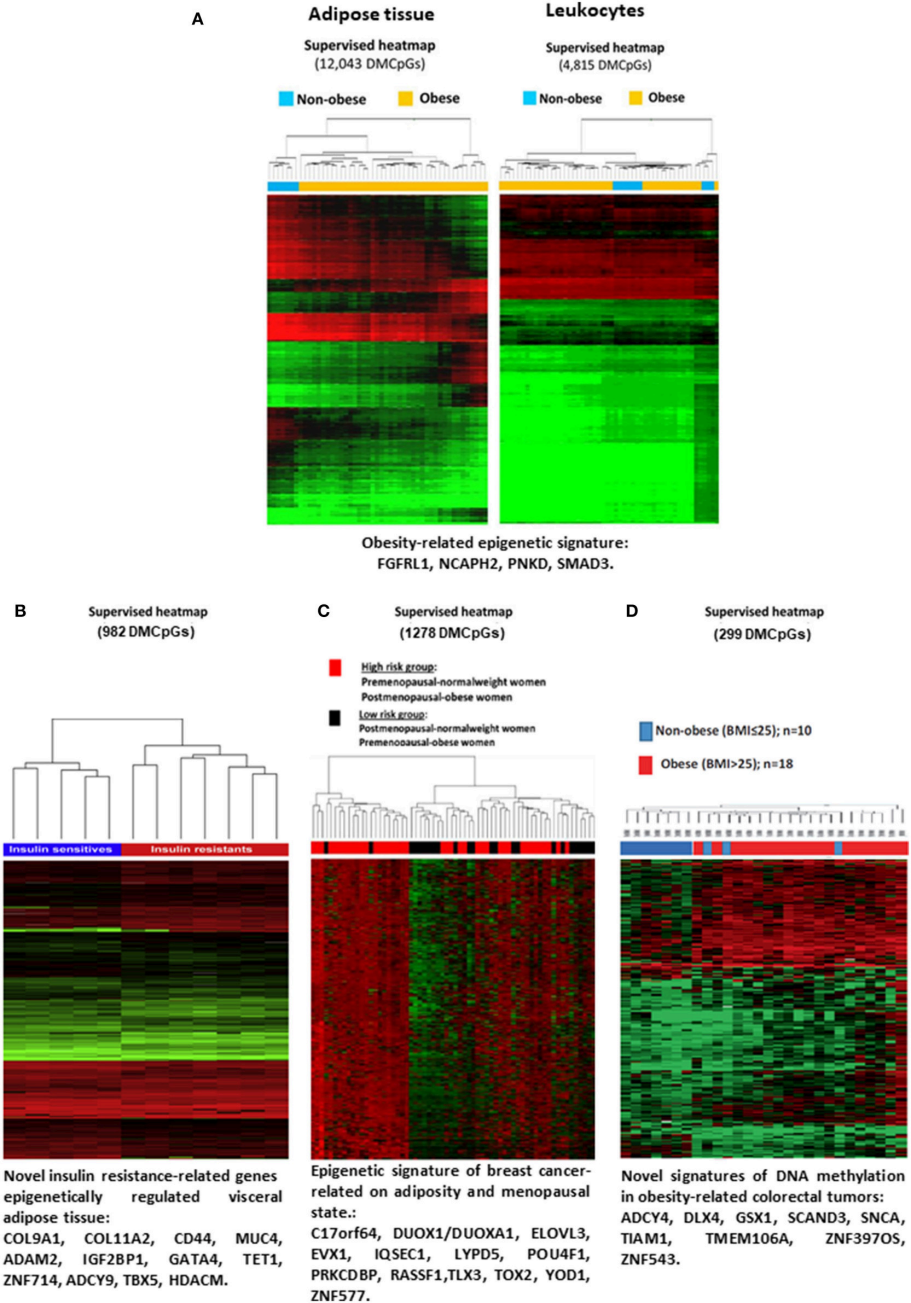
Evidences of DNA methylation associated with obesity and codiseases. **(A)** Heatmap showing differences in the global methylation levels of the overall valid CpGs between the obese and non-obese subcutaneous adipose tissue samples (right panel) and leukocyte samples (left panel). Reprinted with permission from Crujeiras et al. (48). **(B)** Heatmap representing the differential DNA methylation in insulin-resistant and insulin sensitive patients. Reprinted with permission from Crujeiras et al. (49). **(C)** Supervised clustering of the CpGs found differentially methylated in breast tumor samples taking into account menopausal and adiposity status. Reprinted with permission from Crujeiras et al. (50). **(D)** Supervised clustering of the CpGs that were found to be differentially methylated in colorectal tumor samples from the obese and non-obese patients. Reprint with permission from Crujeiras et al. (51). DMCpGs, differentially methylation CpG sites.

In line, our research group conducted a study using isolated DNA samples of subcutaneous adipose tissue and circulating leukocytes obtained from obese and non-obese patients (Figure 2A). This study provided new and valuable biomarkers for the DNA methylation of adipose tissue pathogenesis related to obesity, through peripheral blood analysis (48). There are several other examples regarding the objective of investigating whether the methylation signature of blood cell DNA is capable of reflecting the methylation status of adipose tissue. Specifically, it was determined in one study that the DNA methylation profile in peripheral blood mononuclear cells (PBMC) showed the methylation of white adipose tissue (WAT), with similar mean methylation of CpGs in the subcutaneous adipose and visceral adipose tissues (52). In another recent study, alterations in DNA methylation were identified in several tissues from obese patients, which influenced the pathogenesis of T2D (53).

Regarding the effect of DNA methylation in the promotion of obesity comorbidities, there is an abundance of evidence in the literature. For instance, an epigenetic analysis of the whole genome of the visceral adipose tissue of patients with morbid obesity was performed according to their sensitivity to insulin (49). The results obtained identified an methylome specific of insulin resistance in visceral adipose tissue, in which potential epigenetic biomarkers and new therapeutic targets for the alterations in insulin sensitivity associated with obesity were identified (Figure 2B) (49). In this line, a very recent study from the Methyl Epigenome Network Association (MENA) project evidenced that the methylation levels of 478 CpG sites in blood leukocytes are differentially methylation depending on the HOMA-IR cut off point of 3 units, being the methylation of these differentially methylated CpG sites good predictors of insulin resistance (54). Insulin resistance is the more relevant component of metabolic syndrome and related to this disorder, differential methylation was observed. Both global DNA methylation and the methylation levels of specific genes related to adipose tissue function and metabolism in visceral adipose tissue were found to be related to the metabolic syndrome etiology (55, 56). In blood leukocytes also a specific DNA methylation pattern was found to be related to metabolic syndrome and the difference was consistent especially for ABCG1 gene (57).

Obesity is also related to the development and progression of non-alcoholic fatty liver disease (NAFLD). Moreover, epigenetics was proposed to be a relevant player in the onset of NAFLD. In this regards several differentially methylated regions (DMR) were found depending on the status of liver fibrosis. Importantly, these DMRs were associated with metabolic pathways and were reversed after weight loss (58).

Additionally, in the association between obesity and cancer, it was evidenced that epigenetic regulation, specially DNA methylation, may have a role. A specific methylome in post-menopausal breast cancer has been identified (Figure 2C) (50). Similarly, an analysis of the human colorectal cancer (CRC) methylome was associated with obesity (Figure 2D) (51). Some the BMI-associated CpG sites in blood leukocytes were also found to be related to hepatocellular carcinoma, evidencing the potential effect of obesity on hepatocellular carcinoma mediated by epigenetic mechanisms (59).

### ncRNAs in Obesity

In terms of epigenetic regulation and obesity physiopathology, epigenetic mechanisms other than DNA methylation have also been evaluated. Studies have demonstrated the involvement of ncRNAs, such as miRNAs (60, 61) and lncRNAs (62) in body weight homeostasis and obesity co-morbidities. Emerging evidence suggests that microRNAs play a key role in the pathological development of obesity through their influence of adipocyte differentiation (63). In addition, interesting reports suggest that miRNAs may be regulated by diet and lifestyle factors. Slattery et al. showed some evidence of differences in miRNA expression associated with differences in food composition, even though this study did not find a relationship between miRNA expression and BMI (64). Also, miRNA may respond to various nutritional interventions (65). Moreover, miRNA expression profiles were correlated with subcutaneous adipose tissue and BMI (66). Other studies have aimed to identify the circulating miRNAs related to obesity that could be secreted from adipocytes and explore their possible role in the pathogenesis of metabolic disease (67, 68).

Several lncRNAs regulate adipogenesis (69), and they respond to metabolic transcription factors and hormones and nutrients (70). Three lncRNAs (lncRNA-p5549, lncRNA-p21015, and lncRNA-p19461) were found to be decreased in blood from obese individuals (71). From the identified lncRNAs, the lncRNA-p19461 expression level was found to be significantly increased in eight obese human individuals after a 12-week diet-induced weight-loss therapy, suggesting that circulating lncRNAs are altered in obesity and can be reversed after weight-loss treatment (71). LncRNAs, such as ASMER-1 and ASMER-2, have been proposed as key players in the insulin resistance associated with obesity (72) and as being involved in the potential mechanism in the link between obesity and cancer (73).In short, in recent years, numerous studies have been focusing on epigenetics as a link between environmental factors and a greater predisposition to the development of obesity and its comorbidities (17). This evidence proposes these epigenetic modifications as early prognostic markers for diseases such as obesity, allowing for the individuals stratification depending on the risk for developing a metabolic disorder, and the design of strategies for disease prevention and treatment in a personalized manner (26, 74). This has been demonstrated in studies focusing on epigenetics and precision medicine in obesity, and those on the epigenetic biomarkers of obesity and obesity-related diseases (75–77).

## Changes in Epigenetic Marks After Bariatric Surgery

Bariatric surgery induces several beneficial effects on metabolism apart from weight loss. These beneficial effects lead to improvements in insulin sensitivity, resolving T2D (78). Additionally, after bariatric surgery, obese patients gain cardiovascular function (79). More relevantly, weight loss after bariatric surgery was found to be capable of decreasing the risk of hormone-related cancers in obese patients (80). These beneficial effects induced by weight loss and reduced calorie intake can be promoted by several pathways. It was recently demonstrated that bariatric surgery induces a new metabolic state that appears to be conserved in rodents and humans (81). After bariatric surgery, the chronic low-grade inflammation characteristic of obesity is resolved (82), and the release of incretin hormones is improved (83). Moreover, lipid oxidation and mitochondrial function improve after bariatric surgery (84). However, the molecular mechanism behind this is still unclear. As epigenetic regulation can be a relevant pathway in the expression modulation of the genes involved in the physiology and metabolism in human obesity (85), bariatric surgery could contribute to improvements in the metabolism by means of epigenetic regulation.

Several studies have evaluated the differences in DNA methylation levels after bariatric surgery (86, 87). Global DNA methylation data were inconsistent; however, RYGB surgery was able to modify the DNA methylation level of specific sites (86). For instance, in muscle tissue, it was observed obesity or weight loss induced by RYGB surgery is not associated with global cytosine methylation differences (88). Similarly, the methylation levels of long interspersed nuclear element 1 (LINE-1), a marker of global DNA methylation, did not change after RYGB bariatric surgery-induced weight loss (89, 90). In contrast, the methylation of specific genes underwent changes after weight loss-induced surgery. In muscle, significant changes were observed after RYGB in the DNA methylation profiles of PGC1A and PDK4, in addition to changes in the methylation at the CpG shores and exonic regions close to the transcription start sites. These changes after bariatric surgery were also associated with changes in the transcript levels, suggesting that these weight loss-induced epigenetic changes play a role in the regulation of metabolic gene transcription loss (88). A recent study demonstrated that weight loss induced by RYGB surgery is able to restored to normal levels the SORBS3 methylation and gene expression in the muscle (91). Alterations in the expression and methylation patterns of genes involved in insulin signaling as well as those related to specific non-alcoholic fatty liver disease (NAFLD) were also observed in liver biopsies after bariatric surgery (92). Adipose tissue, the key player in obesity biology, showed significant DNA methylation differences after RYGB bariatric surgery (93). These differences were observed in subcutaneous and omental adipose tissue and were correlated with differences in the transcript levels of differentially methylated genes, suggesting the role of adipose tissue DNA methylation in obesity and the possibility of its remodeling after surgery-induced weight loss (93).

Novel data were also observed when the epigenetic signature of obesity was evaluated in spermatozoa. DNA methylation of central control of appetite genes in sperm varied between obese and non-obese subjects and RYGB surgery-induced weight loss (94).

Apart from target tissue analysis, the methylation profile of specific genes was also evaluated in blood leukocytes before and after bariatric surgery. Although DNA methylation is tissue-specific, blood cells are easily accessible and useful tools for the performance of longitudinal epigenetic studies especially when the target tissue is inaccessible (17). After RYGB operation, the DNA methylation profile of obese individuals changed and resembled that of non-obese patients (95). The differentially methylated promoter was enriched in metabolic processes, demonstrating the potential association between the methylation pattern and beneficial effect of bariatric surgery on metabolic homeostasis. Moreover, the methylation of specific genes belonging to inflammatory pathways, such as SERPINE-1, IL-6, TNFA, IL-1B, and PKD4, was modulated after RYGB surgery (89, 96). A recent study demonstrated that the promoter methylation levels of the NFKB1 gene were significantly increased after surgery, and these changes were associated with changes in the circulating levels of inflammatory markers and blood pressure (97). Additionally, the methylation level of the genes involved in metabolic pathways also showed changes after bariatric surgery. The methylation levels of the stearoyl CoA desaturase-1 (SCD) gene promoter were decreased in morbidly obese individuals before bariatric surgery, and increased 6 months after RYGB, resembling those observed in individuals in the control group (98). SCD is an endoplasmic reticulum-bound enzyme that transforms different saturated fatty acids into monounsaturated fatty acids (99). In fact, this increase was associated with changes in the parameters related to insulin resistance, such as free fatty acid levels, homeostasis model assessment-estimated insulin resistance values, as well as the circulating levels of adiponectin (98).

DNA methylation is the most studied and abundant epigenetic mechanism. However, as mentioned above, other players in the epigenetic regulation exist such as ncRNA and chromatin remodeling. Several miRNAs have been implicated in the control of both insulin signaling and glucose metabolism at multiple levels and their expressions were associated with obesity (100). In line with this, Ortega et al. (101) evaluated the microRNAome in the adipose tissue obtained from morbidly obese patients before and after RYGB. This study demonstrated that 15 miRNAs were differentially expressed after surgery-induced weight loss and these miRNAs were related to cell cycle, development, lipid metabolism and inflammatory response (101). Similarly, after bariatric surgery, changes were observed in the adipocyte-derived exosomal microRNAs in association with improvements in insulin resistance (102). Other studies have evaluated the miRNA expression in serum or plasma. After RYGB, changes in the serum microRNA expression were observed compared to those before surgery, in association with insulin resistance parameters, demonstrating that microRNA expression could play an important and unique role in the effect of RYGB on the improvement of insulin secretion and insulin resistance, apart from the influence of decreased weight and body fat factors (103). A very recent study demonstrated that RYGB altered the circulating microRNAomes in a time-dependent manner, and the expression of 48 circulating microRNAs was significantly different. This study indicated that the circulating microRNAs differentially expressed after bariatric surgery are involved in pathways related to metabolic functions and their expression was correlated to BMI, body weight loss and fasting blood glucose levels (104).

LncRNAs constitute a novel class of RNAs that regulate gene expression; they are involved in the regulation of the expression of several genes belonging to relevant cellular functions. The exploration of their differential expression in relation to metabolism and endocrine regulation requires further investigation for the identification of potential therapeutic targets for obesity (62, 71). Few studies have explored the modulation of the expression of lncRNAs after bariatric surgery, and most of them were performed in animal models. In high-fat diet-induced diabetic mice, a total of 301 lncRNAs, including 232 that were upregulated and 69 that were downregulated, were differentially expressed in the duodenum between the duodenal-jejunal bypass and sham groups (105). These differentially expressed lncRNAs were related to pathways involved in inflammation and insulin secretion. It is suggested that the modulators of NONMMUG021726, the top-ranked gene, have the potential to be used as therapeutic targets or biomarkers for the prediction of bariatric surgery outcomes (105). NONMMUT023781 lncRNA expression was also identified as a potential target for glucose homeostasis after bariatric surgery in the jejune of high-fat diet-induced diabetic mice (106). This study demonstrated the differential expression of 827 jejunal lncRNAs in the duodenal-jejunal bypass group compared to the sham group (105). These dysregulated lncRNAs were related to neuromodulation-related pathways or biological processes, including serotonergic, glutamatergic, and dopaminergic synapses, suggesting the potential involvement of these lncRNA in the remission of T2D via the gut-brain axis (105).

Regarding other epigenetic mechanisms such as chromatin remodeling and histone modifications, no studies till date have focused on the effect of bariatric surgery. Further studies must evaluate the potential effect of bariatric surgery on these epigenetic mechanisms.

Transgenerational studies performed on the offspring of obese women before and after bariatric surgery provide potential evidence of the effect of bariatric surgery on epigenetic mechanisms. It was observed that children born before bariatric surgery show different methylome than children born after surgery, suggesting that the surgical intervention of mothers is able to modify the epigenetic mechanisms of the offspring. The identified genes differentially methylated between before bariatric surgery and after bariatric surgery siblings were related to insulin receptor signaling, type 2 diabetes signaling, and leptin signaling in obesity (107). A previous study demonstrated that the surgical treatment of severe maternal obesity resulted in sustained differences in the methylome and transcriptome of the genes involved in the inflammatory pathways in offspring born after the mothers underwent surgery, compared to their siblings conceived pre-surgery (108). On comparing the methylation profiles of siblings born before and after maternal bypass surgery, the CpG site-encoded genes involved in the glucoregulatory pathways were differentially methylated and potentially responsible for the improved cardiometabolic risk profile with greater insulin sensitivity in siblings conceived post-surgery (109). These studies demonstrate that the improvements in the obesity-related features of mothers following bariatric surgery are reflected in gene methylation and the expression levels of metabolic, immunologic, and inflammatory genes in their offspring. Thus, surgically induced reduction in body weight in a woman of reproductive age can reduce obesity in her children, potentially via epigenetic mechanisms (110).

Other issue that should be considered regarding to the association between the bariatric surgery effect on epigenetic mechanisms is that mediated by the microbiota composition. Bariatric surgery is able to modify the composition of microbiota and part of the beneficial effects of bariatric surgery could be mediated by the composition of microbiota after the surgical intervention and the changes correlate with changes in body composition and glucose homeostasis parameters (111). Importantly, the metabolites derived from microbiota can induce changes in DNA methylation and expression of non-coding-RNA. These changes in epigenetics machinery induced by microbial metabolites were proposed to have potential roles for preventing diseases development such as obesity (112). Further studies are needed to elucidate the crosstalk between microbiota metabolites and epigenetic machinery.

Taken together, the findings of all the aforementioned studies indicate that the beneficial effects induced by bariatric surgery on metabolism, immunity and inflammation can be mediated by epigenetic mechanisms, and also that these beneficial effects induce epigenetic mechanisms that determine descendant phenotype. Most of the studies were performed in small cohorts and therefore, the results have limited translation to the general population. However, the represent a very good start point for future studies in the field.

## Possible Therapeutic Direction in The Future

Relevant topics for discussion include the role of the epigenetic marks induced by bariatric surgery on the prognosis of obesity, as well as the susceptibility to the regain of weight loss. Bariatric surgery is able to induce long-term weight loss and resolves obesity co-morbidities with high efficacy. However, 1–2 years after surgery weight regain may occur (113). As mentioned above, epigenetic marks have been extensively proposed as biomarkers of the development and prognosis of several diseases, including obesity (17). However, few epigenetic studies till date have evaluated the association between the epigenetic marks induced by bariatric surgery and obesity-related prognoses. Only one very recent study focused on epigenomics and bariatric surgery efficacy. Even though it should be verified in an independent study, this work suggests that miR-448 and its target gene SIRT1 could serve as prognostic indicators for obese T2D patients after laparoscopic bariatric surgery (114). That study was performed using peripheral blood obtained from obese T2D patients. Patients were classified according to the clinical remission in response to bariatric surgery into the effective and ineffective groups. The time of efficacy evaluation was 3 months after surgery. Thus, the miR-448 expression was lower and mRNA and protein expression of SIRT1 was higher in the effective group than in the ineffective group after surgery (114). Regarding other epigenetic markers, patients who regained the weight loss induced by nutritional intervention exhibited differential DNA methylation patterns in the subcutaneous adipose tissue (115) or leukocytes (116) compared to patients who were able to maintain their weight loss over a short or long period. No relevant studies in this field have focused on bariatric surgery and obesity prognoses. Longitudinal studies must be conducted to explore the capacity of DNA methylation levels and other epigenetic marks in the prediction of the efficiency of bariatric surgery in the maintenance of weight loss and resolution of obesity co-morbidities, and provide potential therapeutic targets to guarantee the long-term success of bariatric surgery. On the other hand, changes in diet after bariatric surgery could be a relevant cause of changes in the epigenome. Longitudinal studies could also help to elucidate the effect of diet after bariatric surgery on epigenetics and its association with the success of the surgical intervention in counteraction obesity and its comorbidities.

## Conclusions

**Figure 3:**
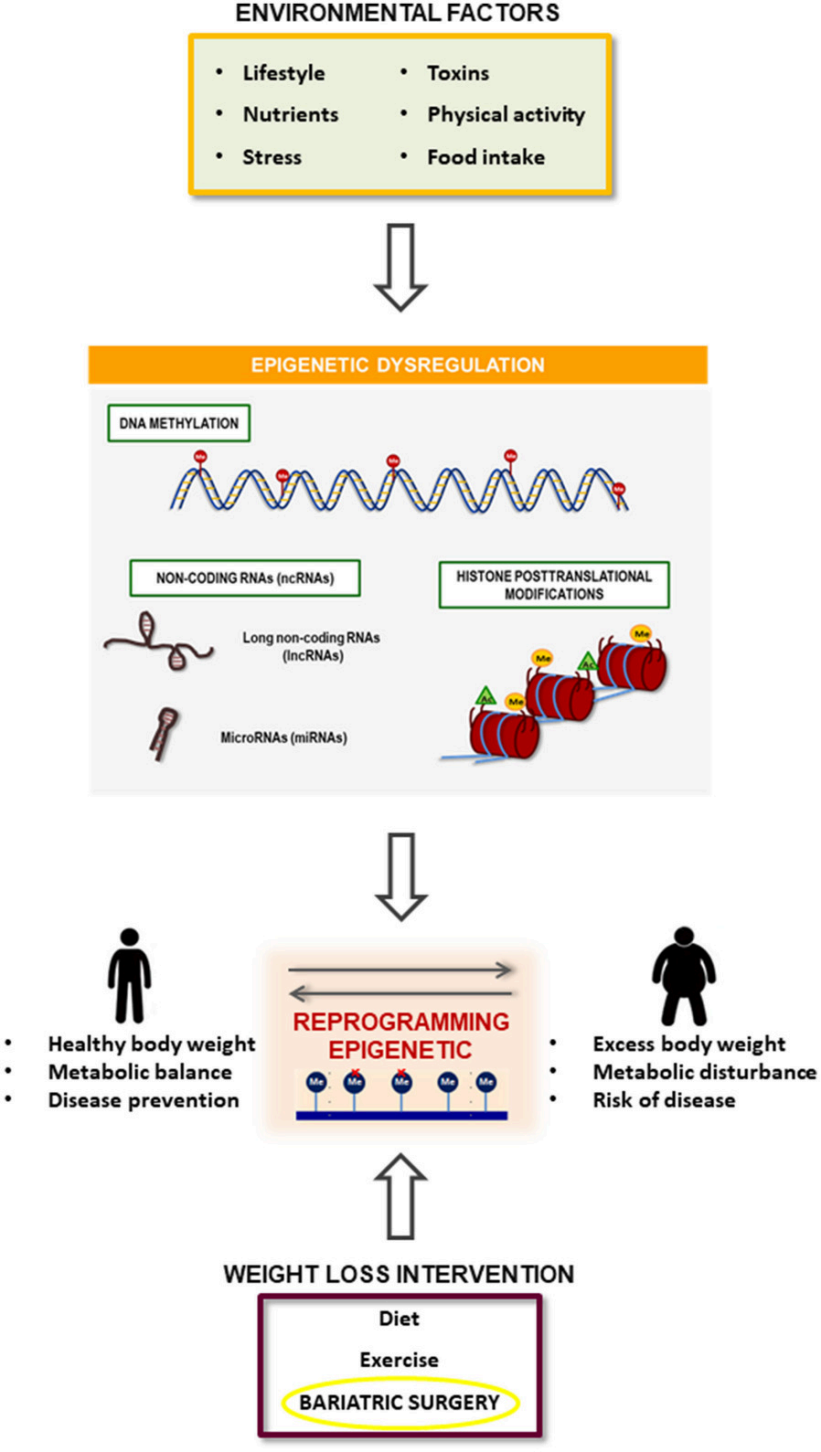
Summary of the relationship between obesity development and bariatric surgery effect mediated by epigenetic mechanisms. Obesity is associated with a specific epigenetic dysregulation that is induced by several environmental factors such as toxics, dietary and physical activity habits and psychological events. On the other hand, bariatric surgery induces an improvement in obesity pathophysiology that could be mediated by the reversion of the obesity-related epigenetic dysregulation.

Considering that obesity features are regulated by epigenetic mechanisms and that bariatric surgery appears to be capable of reversing the obesity-related epigenome, identification of potential epigenetic marks associated with the response to bariatric surgery may open new doors to the personalized therapy for severe obesity. This evidence is relatively new, as epigenetics regulation has been evaluated in the field of obesity only in the past few years, and few studies at present are involved in clarifying the role of epigenetic marks in obesity physiopathology and management. A limited number of studies have demonstrated differential DNA methylation after bariatric surgery and the differential expression of non-coding RNAs (miRNAs and lncRNAs). Epigenetic regulation appears to mediate the beneficial effect of bariatric surgery on body weight and the metabolic disturbances associated with excess body weight, such as insulin resistance, hypertension, cardiovascular disease. It is because bariatric surgery could be able to reprogram or reverse the epigenetic profile associated to obesity (Figure 3). However, regarding the prognosis of obesity after bariatric surgery, there is an urgent need to perform longitudinal studies to evaluate the capacity of epigenetic marks in the prediction of bariatric surgery response. Future interventional and long-term studies are required to further discover the mechanisms and predictive biomarkers of weight regain and inefficiency after bariatric surgery, focusing on epigenetic modifications, and the link between environment and cellular function.

## Author Contributions

AI and AC prepared the first draft of the manuscript. AI was involved in the literature search. AC critically revised and wrote the final version of the manuscript.

### Conflict of Interest Statement

The authors declare that the research was conducted in the absence of any commercial or financial relationships that could be construed as a potential conflict of interest.

## References

[1] Gadde KM Martin CK Berthoud HR Heymsfield SB. Obesity: pathophysiology and management. J Am Coll Cardiol. (2018) 71:69–84. 10.1016/j.jacc.2017.11.011 29301630 PMC7958889

[2] Upadhyay J Farr O Perakakis N Ghaly W Mantzoros C. Obesity as a disease. Med Clin North Am. (2018) 102:13–33. 10.1016/j.mcna.2017.08.004 29156181

[3] Collaboration NCDRF Trends in adult body-mass index in 200 countries from 1975 to 2014: a pooled analysis of 1698 population-based measurement studies with 19.2 million participants. Lancet. (2016) 387:1377–96. 10.1016/S0140-6736(16)30054-X 27115820 PMC7615134

[4] Marti A Martinez-Gonzalez MA Martinez JA. Interaction between genes and lifestyle factors on obesity. Proc Nutr Soc. (2008) 67:1–8. 10.1017/S002966510800596X 18234126

[5] Eckel RH Grundy SM Zimmet PZ. The metabolic syndrome. Lancet. (2005) 365:1415–28. 10.1016/S0140-6736(05)66378-7 15836891

[6] Casanueva FF Moreno B Rodriguez-Azeredo R Massien C Conthe P Formiguera X. Relationship of abdominal obesity with cardiovascular disease, diabetes and hyperlipidaemia in Spain. Clin Endocrinol. (2010) 73:35–40. 10.1111/j.1365-2265.2009.03727.x 19832855

[7] De Mello VD Pulkkinen L Lalli M Kolehmainen M Pihlajamaki J Uusitupa M. DNA methylation in obesity and type 2 diabetes. Ann Med. (2014) 46:103–13. 10.3109/07853890.2013.857259 24779963

[8] Leech RM Mcnaughton SA Timperio A. The clustering of diet, physical activity and sedentary behavior in children and adolescents: a review. Int J Behav Nutr Phys Act. (2014) 11:4. 10.1186/1479-5868-11-4 24450617 PMC3904164

[9] Elder KA Wolfe BM. Bariatric surgery: a review of procedures and outcomes. Gastroenterology. (2007) 132:2253–71. 10.1053/j.gastro.2007.03.057 17498516

[10] Poirier P Cornier MA Mazzone T Stiles S Cummings S Klein S. Bariatric surgery and cardiovascular risk factors: a scientific statement from the American Heart Association. Circulation. (2011) 123:1683–701. 10.1161/CIR.0b013e3182149099 21403092

[11] Velapati SR Shah M Kuchkuntla AR Abu-Dayyeh B Grothe K Hurt RT. Weight regain after bariatric surgery: prevalence, etiology, and treatment. Curr Nutr Rep. (2018) 7:329–34. 10.1007/s13668-018-0243-0 30168043

[12] Waddington CH. Towards a theoretical biology. Nature. (1968) 218:525–7. 10.1038/218525a0 5650959

[13] Wu C Morris JR Genes, genetics, and epigenetics: a correspondence. Science. (2001) 293:1103–5. 10.1126/science.293.5532.1103 11498582

[14] Skinner MK Manikkam M Guerrero-Bosagna C. Epigenetic transgenerational actions of environmental factors in disease etiology. Trends Endocrinol Metab. (2010) 21:214–22. 10.1016/j.tem.2009.12.007 20074974 PMC2848884

[15] Portela A Esteller M. Epigenetic modifications and human disease. Nat Biotechnol. (2010) 28:1057–68. 10.1038/nbt.1685 20944598

[16] Xu W Wang F Yu Z Xin F. Epigenetics and cellular metabolism. Genet Epigenet. (2016) 8:43–51. 10.4137/GEG.S32160 27695375 PMC5038610

[17] Crujeiras AB Diaz-Lagares A DNA methylation in obesity and associated diseases. In: García-Giménez J, editor. Epigenetic Biomarkers and Diagnostics Madrid (2015). p. 313–29. 10.1016/B978-0-12-801899-6.00016-4

[18] Tollefsbol TO An overview of medical epigenetics. In: Tollefsbol T, editor. Medical Epigenetics London (2016). p. 3–7. 10.1016/B978-0-12-803239-8.00001-6

[19] Jones PA. Functions of DNA methylation: islands, start sites, gene bodies and beyond. Nat Rev Genet. (2012) 13:484–92. 10.1038/nrg3230 22641018

[20] Kelly AD Issa JJ. The promise of epigenetic therapy: reprogramming the cancer epigenome. Curr Opin Genet Dev. (2017) 42:68–77. 10.1016/j.gde.2017.03.015 28412585

[21] Coolen MW Statham AL Qu W Campbell MJ Henders AK Montgomery GW. Impact of the genome on the epigenome is manifested in DNA methylation patterns of imprinted regions in monozygotic and dizygotic twins. PLoS ONE. (2011) 6:e25590. 10.1371/journal.pone.0025590 21991322 PMC3184992

[22] Ziech D Franco R Pappa A Panayiotidis MI. Reactive oxygen species (ROS)–induced genetic and epigenetic alterations in human carcinogenesis. Mutat Res. (2011) 711:167–73. 10.1016/j.mrfmmm.2011.02.015 21419141

[23] Milagro FI Gomez-Abellan P Campion J Martinez JA Ordovas JM Garaulet M. CLOCK, PER2 and BMAL1 DNA methylation: association with obesity and metabolic syndrome characteristics and monounsaturated fat intake. Chronobiol Int. (2012) 29:1180–94. 10.3109/07420528.2012.719967 23003921

[24] Yong WS Hsu FM Chen PY. Profiling genome-wide DNA methylation. Epigenetics Chromatin. (2016) 9:26. 10.1186/s13072-016-0075-3 27358654 PMC4926291

[25] Estey EH. Epigenetics in clinical practice: the examples of azacitidine and decitabine in myelodysplasia and acute myeloid leukemia. Leukemia. (2013) 27:1803–12. 10.1038/leu.2013.173 23757301

[26] Milagro FI Mansego ML De Miguel C Martinez JA. Dietary factors, epigenetic modifications and obesity outcomes: progresses and perspectives. Mol Aspects Med. (2013) 34:782–812. 10.1016/j.mam.2012.06.010 22771541

[27] Mikeska T Craig JM. DNA methylation biomarkers: cancer and beyond. Genes. (2014) 5:821–64. 10.3390/genes5030821 25229548 PMC4198933

[28] Agardh E Lundstig A Perfilyev A Volkov P Freiburghaus T Lindholm E. Genome-wide analysis of DNA methylation in subjects with type 1 diabetes identifies epigenetic modifications associated with proliferative diabetic retinopathy. BMC Med. (2015) 13:182. 10.1186/s12916-015-0421-5 26248552 PMC4527111

[29] Lisanti S Omar WA Tomaszewski B De Prins S Jacobs G Koppen G. Comparison of methods for quantification of global DNA methylation in human cells and tissues. PLoS ONE. (2013) 8:e79044. 10.1371/journal.pone.0079044 24260150 PMC3832524

[30] Laird PW. Principles and challenges of genomewide DNA methylation analysis. Nat Rev Genet. (2010) 11:191–203. 10.1038/nrg2732 20125086

[31] Cokus SJ Feng S Zhang X Chen Z Merriman B Haudenschild CD. Shotgun bisulphite sequencing of the Arabidopsis genome reveals DNA methylation patterning. Nature. (2008) 452:215–9. 10.1038/nature06745 18278030 PMC2377394

[32] Hanna CW Demond H Kelsey G. Epigenetic regulation in development: is the mouse a good model for the human? Hum Reprod Update. (2018) 24:556–76. 10.1093/humupd/dmy021 29992283 PMC6093373

[33] Egger G Liang G Aparicio A Jones PA. Epigenetics in human disease and prospects for epigenetic therapy. Nature. (2004) 429:457–63. 10.1038/nature02625 15164071

[34] Steger DJ Grant GR Schupp M Tomaru T Lefterova MI Schug J. Propagation of adipogenic signals through an epigenomic transition state. Genes Dev. (2010) 24:1035–44. 10.1101/gad.1907110 20478996 PMC2867208

[35] Okuno Y Ohtake F Igarashi K Kanno J Matsumoto T Takada I. Epigenetic regulation of adipogenesis by PHF2 histone demethylase. Diabetes. (2013) 62:1426–34. 10.2337/db12-0628 23274892 PMC3636657

[36] Nie L Shuai L Zhu M Liu P Xie ZF Jiang S. The landscape of histone modifications in a high-fat Diet-Induced Obese (DIO) mouse model. Mol Cell Proteomics. (2017) 16:1324–34. 10.1074/mcp.M117.067553 28450421 PMC5500764

[37] Abu-Farha M Tiss A Abubaker J Khadir A Al-Ghimlas F Al-Khairi I. Proteomics analysis of human obesity reveals the epigenetic factor HDAC4 as a potential target for obesity. PLoS ONE. (2013) 8:e75342. 10.1371/journal.pone.0075342 24086512 PMC3782461

[38] Taft RJ Pang KC Mercer TR Dinger M Mattick JS. Non-coding RNAs: regulators of disease. J Pathol. (2010) 220:126–39. 10.1002/path.2638 19882673

[39] Macfarlane LA Murphy PR. microRNA: biogenesis, function and role in cancer. Curr Genomics. (2010) 11:537–61. 10.2174/138920210793175895 21532838 PMC3048316

[40] Peng Y Yu S Li H Xiang H Peng J Jiang S. microRNAs: emerging roles in adipogenesis and obesity. Cell Signal. (2014) 26:1888–96. 10.1016/j.cellsig.2014.05.006 24844591

[41] Zaiou M El Amri H Bakillah A. The clinical potential of adipogenesis and obesity-related microRNAs. Nutr Metab Cardiovasc Dis. (2018) 28:91–111. 10.1016/j.numecd.2017.10.015 29170059

[42] Fang Y Fullwood MJ. Roles, functions, and mechanisms of long non-coding RNAs in cancer. Genom Proteom Bioinf. (2016) 14:42–54. 10.1016/j.gpb.2015.09.006 26883671 PMC4792843

[43] Wei S Du M Jiang Z Hausman GJ Zhang L Dodson MV Long non-coding RNAs in regulating adipogenesis: new RNAs shed lights on obesity. Cell Mol Life Sci. (2016) 73:2079–87. 10.1007/s00018-016-2169-2 26943803 PMC5737903

[44] Bays H Scinta W. Adiposopathy and epigenetics: an introduction to obesity as a transgenerational disease. Curr Med Res Opin. (2015) 31:2059–69. 10.1185/03007995.2015.1087983 26331354

[45] Casanello P Krause BJ Castro-Rodriguez JA Uauy R. Epigenetics and obesity. Rev Chil Pediatr. (2016) 87:335–42. 10.1016/j.rchipe.2016.08.009 27692574

[46] Dick KJ Nelson CP Tsaprouni L Sandling JK Aissi D Wahl S. DNA methylation and body-mass index: a genome-wide analysis. Lancet. (2014) 383:1990–8. 10.1016/S0140-6736(13)62674-4 24630777

[47] Van Dijk SJ Tellam RL Morrison JL Muhlhausler BS Molloy PL. Recent developments on the role of epigenetics in obesity and metabolic disease. Clin Epigenetics. (2015) 7:66. 10.1186/s13148-015-0101-5 27408648 PMC4940755

[48] Crujeiras AB Diaz-Lagares A Sandoval J Milagro FI Navas-Carretero S Carreira MC. DNA methylation map in circulating leukocytes mirrors subcutaneous adipose tissue methylation pattern: a genome-wide analysis from non-obese and obese patients. Sci Rep. (2017) 7:41903. 10.1038/srep41903 28211912 PMC5314866

[49] Crujeiras AB Diaz-Lagares A Moreno-Navarrete JM Sandoval J Hervas D Gomez A. Genome-wide DNA methylation pattern in visceral adipose tissue differentiates insulin-resistant from insulin-sensitive obese subjects. Transl Res. (2016) 178:13–24 e15. 10.1016/j.trsl.2016.07.002 27477082

[50] Crujeiras AB Diaz-Lagares A Stefansson OA Macias-Gonzalez M Sandoval J Cueva J. Obesity and menopause modify the epigenomic profile of breast cancer. Endocr Relat Cancer. (2017b) 24:351–63. 10.1530/ERC-16-0565 28442560

[51] Crujeiras AB Morcillo S Diaz-Lagares A Sandoval J Castellano-Castillo D Torres E. Identification of an episignature of human colorectal cancer associated with obesity by genome-wide DNA methylation analysis. Int J Obes. (2018) 43:176–88. 10.1038/s41366-018-0065-6 29717273

[52] Arner P Sahlqvist AS Sinha I Xu H Yao X Waterworth D The epigenetic signature of systemic insulin resistance in obese women. Diabetologia. (2016) 59:2393–405. 10.1007/s00125-016-4074-5 27535281 PMC5506095

[53] Barajas-Olmos F Centeno-Cruz F Zerrweck C Imaz-Rosshandler I Martinez-Hernandez A Cordova EJ. Altered DNA methylation in liver and adipose tissues derived from individuals with obesity and type 2 diabetes. BMC Med Genet. (2018) 19:28. 10.1186/s12881-018-0542-8 29466957 PMC5822594

[54] Arpon A Milagro FI Ramos-Lopez O Mansego ML Santos JL Riezu-Boj JI. Epigenome-wide association study in peripheral white blood cells involving insulin resistance. Sci Rep. (2019) 9:2445. 10.1038/s41598-019-38980-2 30792424 PMC6385280

[55] Castellano-Castillo D Moreno-Indias I Fernandez-Garcia JC Alcaide-Torres J Moreno-Santos I Ocana L. Adipose tissue LPL methylation is associated with triglyceride concentrations in the metabolic syndrome. Clin Chem. (2018) 64:210–8. 10.1373/clinchem.2017.277921 29046332

[56] Castellano-Castillo D Moreno-Indias I Sanchez-Alcoholado L Ramos-Molina B Alcaide-Torres J Morcillo S. Altered adipose tissue DNA methylation status in metabolic syndrome: relationships between global DNA methylation and specific methylation at adipogenic, lipid metabolism and inflammatory candidate genes and metabolic variables. J Clin Med. (2019) 8:87. 10.3390/jcm8010087 30642114 PMC6352101

[57] Akinyemiju T Do AN Patki A Aslibekyan S Zhi D Hidalgo B. Epigenome-wide association study of metabolic syndrome in African-American adults. Clin Epigenetics. (2018) 10:49. 10.1186/s13148-018-0483-2 29643945 PMC5891946

[58] Hotta K Kitamoto A Kitamoto T Ogawa Y Honda Y Kessoku T Identification of differentially methylated region (DMR) networks associated with progression of non-alcoholic fatty liver disease. Sci Rep. (2018) 8:13567 10.1038/s41598-018-31886-5 30206277 PMC6134034

[59] Ramos-Lopez O Riezu-Boj JI Milagro FI Alfredo Martinez J Project M Association of methylation signatures at hepatocellular carcinoma pathway genes with adiposity and insulin resistance phenotypes. Nutr Cancer. (2018) 20:1–12. 10.1080/01635581.2018.1531136 30457363

[60] Heneghan HM Miller N Kerin MJ. Role of microRNAs in obesity and the metabolic syndrome. Obes Rev. (2010) 11:354–61. 10.1111/j.1467-789X.2009.00659.x 19793375

[61] Dumortier O Hinault C Van Obberghen E. microRNAs and metabolism crosstalk in energy homeostasis. Cell Metab. (2013) 18:312–24. 10.1016/j.cmet.2013.06.004 23850315

[62] Giroud M Scheideler M. Long non-coding RNAs in metabolic organs and energy homeostasis. Int J Mol Sci. (2017) 18:E2578. 10.3390/ijms18122578 29189723 PMC5751181

[63] Lin Q Gao Z Alarcon RM Ye J Yun Z. A role of miR-27 in the regulation of adipogenesis. FEBS J. (2009) 276:2348–58. 10.1111/j.1742-4658.2009.06967.x 19348006 PMC5330386

[64] Slattery ML Herrick JS Mullany LE Stevens JR Wolff RK. Diet and lifestyle factors associated with miRNA expression in colorectal tissue. Pharmgenomics Pers Med. (2017) 10:1–16. 10.2147/PGPM.S117796 28053552 PMC5189704

[65] Palmer JD Soule BP Simone BA Zaorsky NG Jin L Simone NL. microRNA expression altered by diet: can food be medicinal? Ageing Res Rev. (2014) 17:16–24. 10.1016/j.arr.2014.04.005 24833329

[66] Kloting N Berthold S Kovacs P Schon MR Fasshauer M Ruschke K. microRNA expression in human omental and subcutaneous adipose tissue. PLoS ONE. (2009) 4:e4699. 10.1371/journal.pone.0004699 19259271 PMC2649537

[67] Wang YC Li Y Wang XY Zhang D Zhang H Wu Q. Circulating miR-130b mediates metabolic crosstalk between fat and muscle in overweight/obesity. Diabetologia. (2013) 56:2275–85. 10.1007/s00125-013-2996-8 23868745

[68] Doumatey AP He WJ Gaye A Lei L Zhou J Gibbons GH. Circulating MiR-374a-5p is a potential modulator of the inflammatory process in obesity. Sci Rep. (2018) 8:7680. 10.1038/s41598-018-26065-5 29769661 PMC5955981

[69] Sun L Goff LA Trapnell C Alexander R Lo KA Hacisuleyman E Long non-coding RNAs regulate adipogenesis. Proc Natl Acad Sci USA. (2013) 110:3387–92. 10.1073/pnas.1222643110 23401553 PMC3587215

[70] Yang L Li P Yang W Ruan X Kiesewetter K Zhu J. Integrative transcriptome analyses of metabolic responses in mice define pivotal LncRNA metabolic regulators. Cell Metab. (2016) 24:627–39. 10.1016/j.cmet.2016.08.019 27667668 PMC5181118

[71] Sun J Ruan Y Wang M Chen R Yu N Sun L. Differentially expressed circulating LncRNAs and mRNA identified by microarray analysis in obese patients. Sci Rep. (2016) 6:35421. 10.1038/srep35421 27767123 PMC5073332

[72] Gao H Kerr A Jiao H Hon CC Ryden M Dahlman I. Long non-coding RNAs associated with metabolic traits in human white adipose tissue. EBioMed. (2018) 30:248–60. 10.1016/j.ebiom.2018.03.010 29580841 PMC5952343

[73] Yau MY Xu L Huang CL Wong CM. Long non-coding RNAs in obesity-induced cancer. Noncoding RNA. (2018) 4:E19. 10.3390/ncrna4030019 30154386 PMC6162378

[74] Martinez JA Milagro FI Claycombe KJ Schalinske KL. Epigenetics in adipose tissue, obesity, weight loss, and diabetes. Adv Nutr. (2014) 5:71–81. 10.3945/an.113.004705 24425725 PMC3884103

[75] Fall T Mendelson M Speliotes EK. recent advances in human genetics and epigenetics of adiposity: pathway to precision medicine? Gastroenterology. (2017) 152:1695–706. 10.1053/j.gastro.2017.01.054 28214526 PMC5576453

[76] Campanella G Gunter MJ Polidoro S Krogh V Palli D Panico S. Epigenome-wide association study of adiposity and future risk of obesity-related diseases. Int J Obes. (2018) 42:2022–35. 10.1038/s41366-018-0064-7 29713043

[77] Cheng Z Zheng L Almeida FA. Epigenetic reprogramming in metabolic disorders: nutritional factors and beyond. J Nutr Biochem. (2018) 54:1–10. 10.1016/j.jnutbio.2017.10.004 29154162 PMC5866737

[78] Schauer PR Mingrone G Ikramuddin S Wolfe B. Clinical outcomes of metabolic surgery: efficacy of glycemic control, weight loss, and remission of diabetes. Diabetes Care. (2016) 39:902–11. 10.2337/dc16-0382 27222548 PMC5864131

[79] Benotti PN Wood GC Carey DJ Mehra VC Mirshahi T Lent MR. Gastric bypass surgery produces a durable reduction in cardiovascular disease risk factors and reduces the long-term risks of congestive heart failure. J Am Heart Assoc. (2017) 6:e005126. 10.1161/JAHA.116.005126 28536154 PMC5524077

[80] Mackenzie H Markar SR Askari A Faiz O Hull M Purkayastha S. Obesity surgery and risk of cancer. Br J Surg. (2018) 105:1650–7. 10.1002/bjs.10914 30003539

[81] Ben-Zvi D Meoli L Abidi WM Nestoridi E Panciotti C Castillo E. Time-dependent molecular responses differ between gastric bypass and dieting but are conserved across species. Cell Metab. (2018) 28:310–23.e6. 10.1016/j.cmet.2018.06.004 30043755 PMC6628900

[82] Zhang C Zhang J Liu Z Zhou Z. More than an anti-diabetic bariatric surgery, metabolic surgery alleviates systemic and local inflammation in obesity. Obes Surg. (2018) 28:3658–68. 10.1007/s11695-018-3400-z 30187424

[83] Ma J Vella A. What has bariatric surgery taught us about the role of the upper gastrointestinal tract in the regulation of postprandial glucose metabolism? Front Endocrinol. (2018) 9:324. 10.3389/fendo.2018.00324 29997575 PMC6028568

[84] Fernstrom M Bakkman L Loogna P Rooyackers O Svensson M Jakobsson T. Improved muscle mitochondrial capacity following gastric bypass surgery in obese subjects. Obes Surg. (2016) 26:1391–7. 10.1007/s11695-015-1932-z 26471784

[85] Van Dijk SJ Molloy PL Varinli H Morrison JL Muhlhausler BS, Members of EpiSCOPE. Epigenetics and human obesity. Int J Obes. (2015) 39:85–97. 10.1038/ijo.2014.34 24566855

[86] Morcillo S Macias-Gonzalez M Tinahones FJ. The effect of metabolic and bariatric surgery on DNA methylation patterns. Curr Atheroscler Rep. (2017) 19:40. 10.1007/s11883-017-0676-8 28853041

[87] Sala P De Miranda Torrinhas RSM Fonseca DC Ravacci GR Waitzberg DL Giannella-Neto D. Tissue-specific methylation profile in obese patients with type 2 diabetes before and after Roux-en-Y gastric bypass. Diabetol Metab Syndr. (2017) 9:15. 10.1186/s13098-017-0214-4 28250848 PMC5322591

[88] Barres R Kirchner H Rasmussen M Yan J Kantor FR Krook A. Weight loss after gastric bypass surgery in human obesity remodels promoter methylation. Cell Rep. (2013) 3:1020–7. 10.1016/j.celrep.2013.03.018 23583180

[89] Nicoletti CF Nonino CB De Oliveira BA Pinhel MA Mansego ML Milagro FI DNA methylation and hydroxymethylation levels in relation to two weight loss strategies: energy-restricted diet or bariatric surgery. Obes Surg. (2016) 26:603–11. 10.1007/s11695-015-1802-8 26198618

[90] Martin-Nunez GM Cabrera-Mulero A Alcaide-Torres J Garcia-Fuentes E Tinahones FJ Morcillo S No effect of different bariatric surgery procedures on LINE-1 DNA methylation in diabetic and non-diabetic morbidly obese patients. Surg Obes Relat Dis. (2017) 13:442–50. 10.1016/j.soard.2016.10.014 27986580

[91] Day SE Garcia LA Coletta RL Campbell LE Benjamin TR De Filippis EA. Alterations of sorbin and SH3 domain containing 3 (SORBS3) in human skeletal muscle following Roux-en-Y gastric bypass surgery. Clin Epigenetics. (2017) 9:96. 10.1186/s13148-017-0396-5 28883895 PMC5581422

[92] Ahrens M Ammerpohl O Von Schonfels W Kolarova J Bens S Itzel T DNA methylation analysis in non-alcoholic fatty liver disease suggests distinct disease-specific and remodeling signatures after bariatric surgery. Cell Metab. (2013) 18:296–302. 10.1016/j.cmet.2013.07.004 23931760

[93] Benton MC Johnstone A Eccles D Harmon B Hayes MT Lea RA. An analysis of DNA methylation in human adipose tissue reveals differential modification of obesity genes before and after gastric bypass and weight loss. Genome Biol. (2015) 16:8. 10.1186/s13059-014-0569-x 25651499 PMC4301800

[94] Donkin I Versteyhe S Ingerslev LR Qian K Mechta M Nordkap L. Obesity and bariatric surgery drive epigenetic variation of spermatozoa in humans. Cell Metab. (2016) 23:369–78. 10.1016/j.cmet.2015.11.004 26669700

[95] Nilsson EK Ernst B Voisin S Almen MS Benedict C Mwinyi J. Roux-en Y gastric bypass surgery induces genome-wide promoter-specific changes in DNA methylation in whole blood of obese patients. PLoS ONE. (2015) 10:e0115186. 10.1371/journal.pone.0115186 25710379 PMC4340013

[96] Kirchner H Nylen C Laber S Barres R Yan J Krook A. Altered promoter methylation of PDK4, IL1 B, IL6, and TNF after Roux-en Y gastric bypass. Surg Obes Relat Dis. (2014) 10:671–8. 10.1016/j.soard.2013.12.019 24837562

[97] Macias-Gonzalez M Martin-Nunez GM Garrido-Sanchez L Garcia-Fuentes E Tinahones FJ Morcillo S. Decreased blood pressure is related to changes in NF-kB promoter methylation levels after bariatric surgery. Surg Obes Relat Dis. (2018) 14:1327–34. 10.1016/j.soard.2018.06.011 30057095

[98] Morcillo S Martin-Nunez GM Garcia-Serrano S Gutierrez-Repiso C Rodriguez-Pacheco F Valdes S. Changes in SCD gene DNA methylation after bariatric surgery in morbidly obese patients are associated with free fatty acids. Sci Rep. (2017) 7:46292. 10.1038/srep46292 28393901 PMC5385880

[99] Ntambi JM Miyazaki M. Regulation of stearoyl-CoA desaturases and role in metabolism. Prog Lipid Res. (2004) 43:91–104. 10.1016/S0163-7827(03)00039-0 14654089

[100] Arner P Kulyte A. microRNA regulatory networks in human adipose tissue and obesity. Nat Rev Endocrinol. (2015) 11:276–88. 10.1038/nrendo.2015.25 25732520

[101] Ortega FJ Mercader JM Moreno-Navarrete JM Nonell L Puigdecanet E Rodriquez-Hermosa JI. Surgery-induced weight loss is associated with the downregulation of genes targeted by microRNAs in adipose tissue. J Clin Endocrinol Metab. (2015) 100:E1467–76. 10.1210/jc.2015-2357 26252355

[102] Hubal MJ Nadler EP Ferrante SC Barberio MD Suh JH Wang J. Circulating adipocyte-derived exosomal microRNAs associated with decreased insulin resistance after gastric bypass. Obesity. (2017) 25:102–10. 10.1002/oby.21709 27883272 PMC5182153

[103] Lirun K Sewe M Yong W. A pilot study: the effect of Roux-en-Y gastric bypass on the serum microRNAs of the type 2 diabetes patient. Obes Surg. (2015) 25:2386–92. 10.1007/s11695-015-1711-x 26138690

[104] Alkandari A Ashrafian H Sathyapalan T Sedman P Darzi A Holmes E. Improved physiology and metabolic flux after Roux-en-Y gastric bypass is associated with temporal changes in the circulating microRNAome: a longitudinal study in humans. BMC Obes. (2018) 5:20. 10.1186/s40608-018-0199-z 29881628 PMC5984421

[105] Liang Y Yu B Wang Y Qiao Z Cao T Zhang P Duodenal long non-coding RNAs are associated with glycemic control after bariatric surgery in high-fat diet-induced diabetic mice. Surg Obes Relat Dis. (2017) 13:1212–26. 10.1016/j.soard.2017.02.010 28366671

[106] Liang Y Yu B Wang Y Qiao Z Cao T Zhang P Jejunal long non-coding RNAs are associated with glycemic control via gut-brain axis after bariatric surgery in diabetic mice. Surg Obes Relat Dis. (2018) 14:821–32. 10.1016/j.soard.2018.03.006 29631984

[107] Berglind D Muller P Willmer M Sinha I Tynelius P Naslund E. Differential methylation in inflammation and type 2 diabetes genes in siblings born before and after maternal bariatric surgery. Obesity. (2016) 24:250–61. 10.1002/oby.21340 26637991

[108] Guenard F Tchernof A Deshaies Y Cianflone K Kral JG Marceau P. Methylation and expression of immune and inflammatory genes in the offspring of bariatric bypass surgery patients. J Obes. (2013) 2013:492170. 10.1155/2013/492170 23840945 PMC3693160

[109] Guenard F Deshaies Y Cianflone K Kral JG Marceau P Vohl MC. Differential methylation in glucoregulatory genes of offspring born before vs. after maternal gastrointestinal bypass surgery. Proc Natl Acad Sci USA. (2013) 110:11439–44. 10.1073/pnas.1216959110 23716672 PMC3710842

[110] Patti ME. Reducing maternal weight improves offspring metabolism and alters (or modulates) methylation. Proc Natl Acad Sci USA. (2013) 110:12859–60. 10.1073/pnas.1309724110 23884649 PMC3740829

[111] Wang FG Bai RX Yan WM Yan M Dong LY Song MM. Differential composition of gut microbiota among healthy volunteers, morbidly obese patients and post-bariatric surgery patients. Exp Ther Med. (2019) 17:2268–78. 10.3892/etm.2019.7200 30867711 PMC6395995

[112] Cuevas-Sierra A Ramos-Lopez O Riezu-Boj JI Milagro FI Martinez JA. Diet, gut microbiota, and obesity: links with host genetics and epigenetics and potential applications. Adv Nutr. (2019) 10:S17–30. 10.1093/advances/nmy078 30721960 PMC6363528

[113] Shantavasinkul PC Omotosho P Corsino L Portenier D Torquati A. Predictors of weight regain in patients who underwent Roux-en-Y gastric bypass surgery. Surg Obes Relat Dis. (2016) 12:1640–5. 10.1016/j.soard.2016.08.028 27989521

[114] Wang Y Wang DS Cheng YS Jia BL Yu G Yin XQ. Expression of microRNA-448 and SIRT1 and prognosis of obese type 2 diabetic mellitus patients after laparoscopic bariatric surgery. Cell Physiol Biochem. (2018) 45:935–50. 10.1159/000487287 29428938

[115] Bollepalli S Kaye S Heinonen S Kaprio J Rissanen A Virtanen KA. Subcutaneous adipose tissue gene expression and DNA methylation respond to both short- and long-term weight loss. Int J Obes. (2018) 42:412–23. 10.1038/ijo.2017.245 28978976

[116] Crujeiras AB Campion J Diaz-Lagares A Milagro FI Goyenechea E Abete I. Association of weight regain with specific methylation levels in the NPY and POMC promoters in leukocytes of obese men: a translational study. Regul Pept. (2013) 186:1–6. 10.1016/j.regpep.2013.06.012 23831408

